# Supplementary figures and images for: Comparative analysis and characterization of the gut microbiota of four farmed snakes from southern China
Source: PeerJ. 2019 Mar 29;7:e6658. doi: 10.7717/peerj.6658 (PMC6442672; doi:10.7717/peerj.6658)

(A)

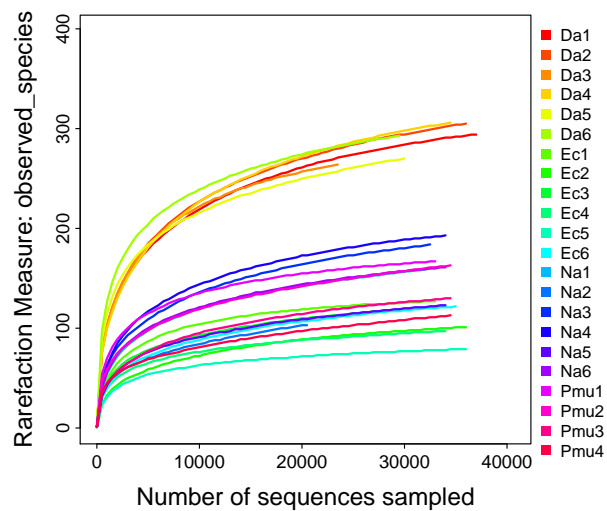

(B)

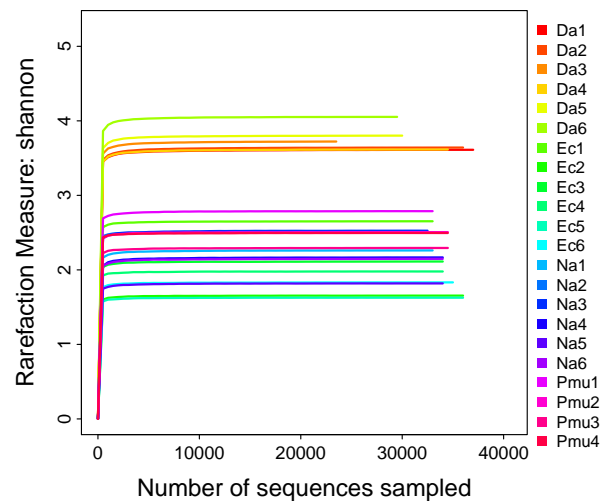

Supplement: Supplemental Information 1 — Da1-Da6 represent the samples collected from the Deinagkistrodon acutus, Ec1-Ec6 represent the samples collected from the Elaphe carinata, Na1-Na6 represent the samples collected from the Naja atra, Pmu1-Pmu6 represent the samples collected from the Ptyas mucosus. [file peerj-07-6658-s001.pdf]

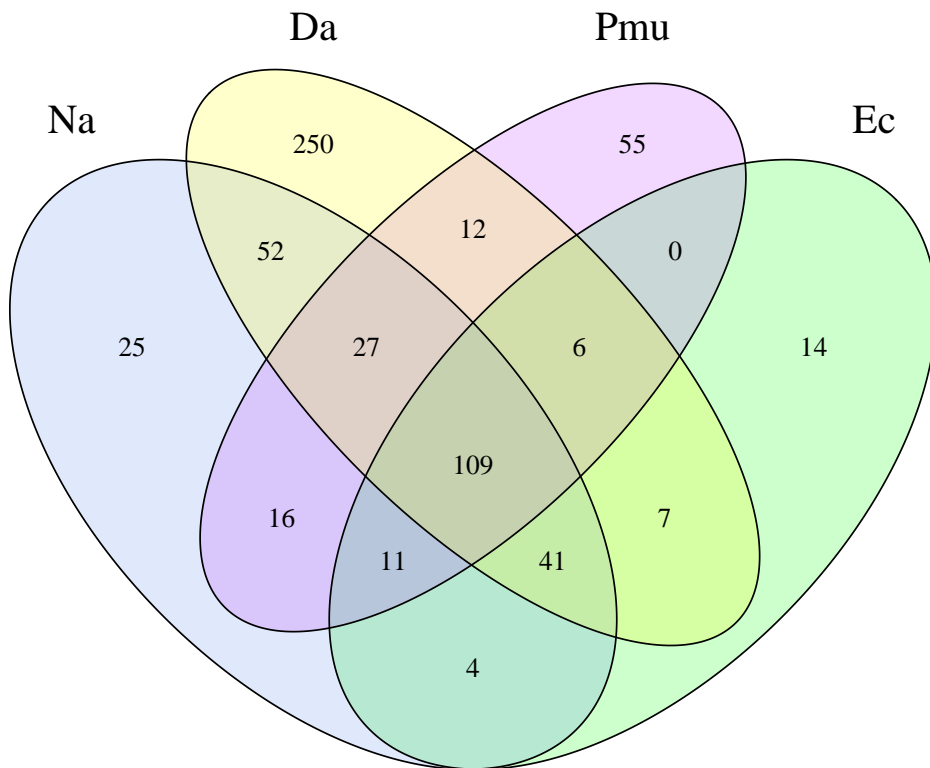

Supplement: Supplemental Information 2 — Different color represents different groups. The interior of each circle symbolically represents the number of observed OTUs in the certain group. The overlapping area or intersection would represent the set of OTU commonly present in the counterpart groups. Likewise, the single-layer zone represents the number of OTUs uniquely found in the certain group. Na, Naja atra group; Pmu, Ptyas mucosus group; Ec, Elaphe carinata group; Da, Deinagkistrodon acutus group. [file peerj-07-6658-s002.pdf]

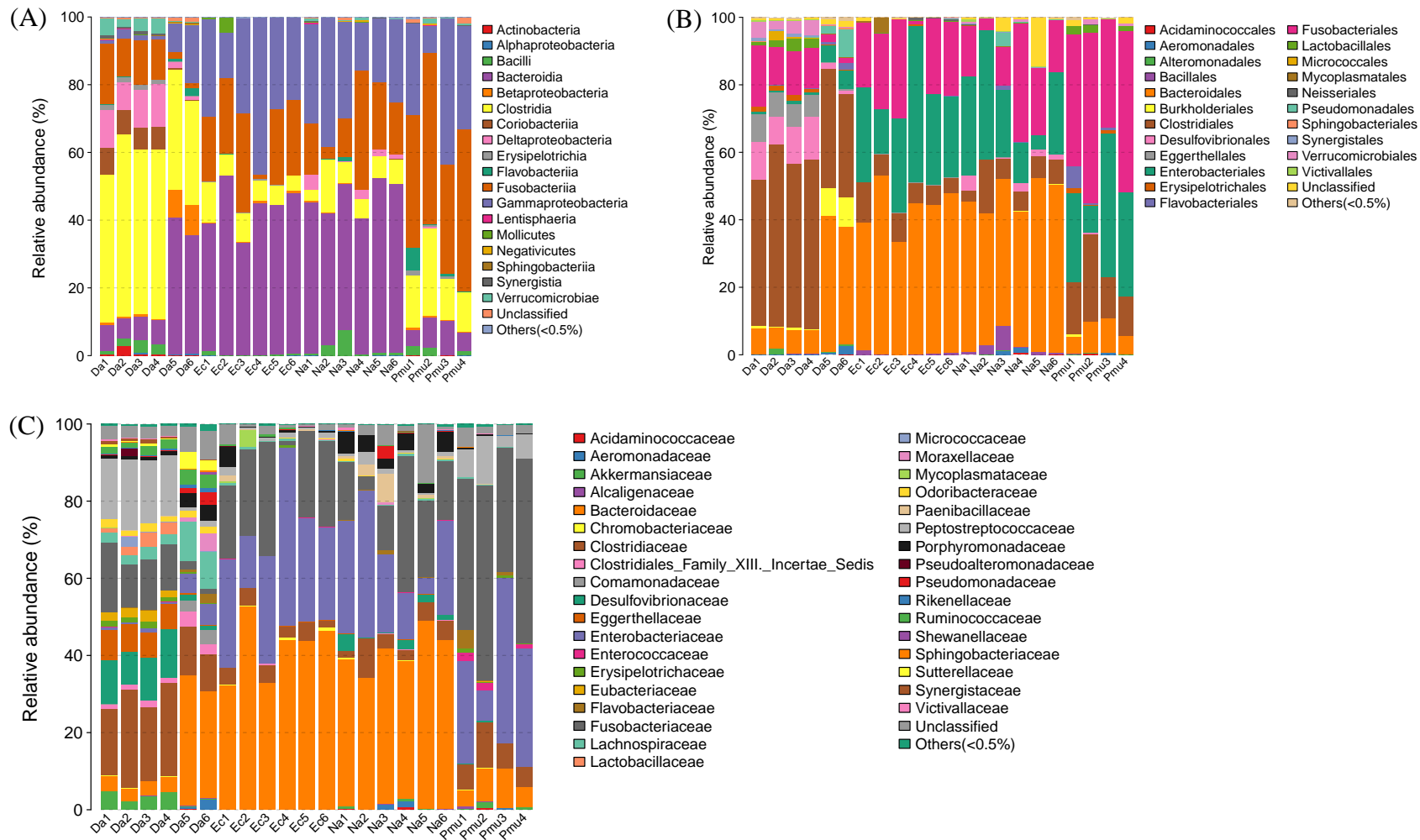

Supplement: Supplemental Information 3 — The ratio of each species in certain sample is directly displayed. The species of which abundance is less than 0.5% in all samples were classified into “others” in other ranks. Na1-Na6, Naja atra group; Pmu1-Pmu4, Ptyas mucosus group; Ec1-Ec6, Elaphe carinata group; Da1-Da6, Deinagkistrodon acutus group. [file peerj-07-6658-s003.pdf]

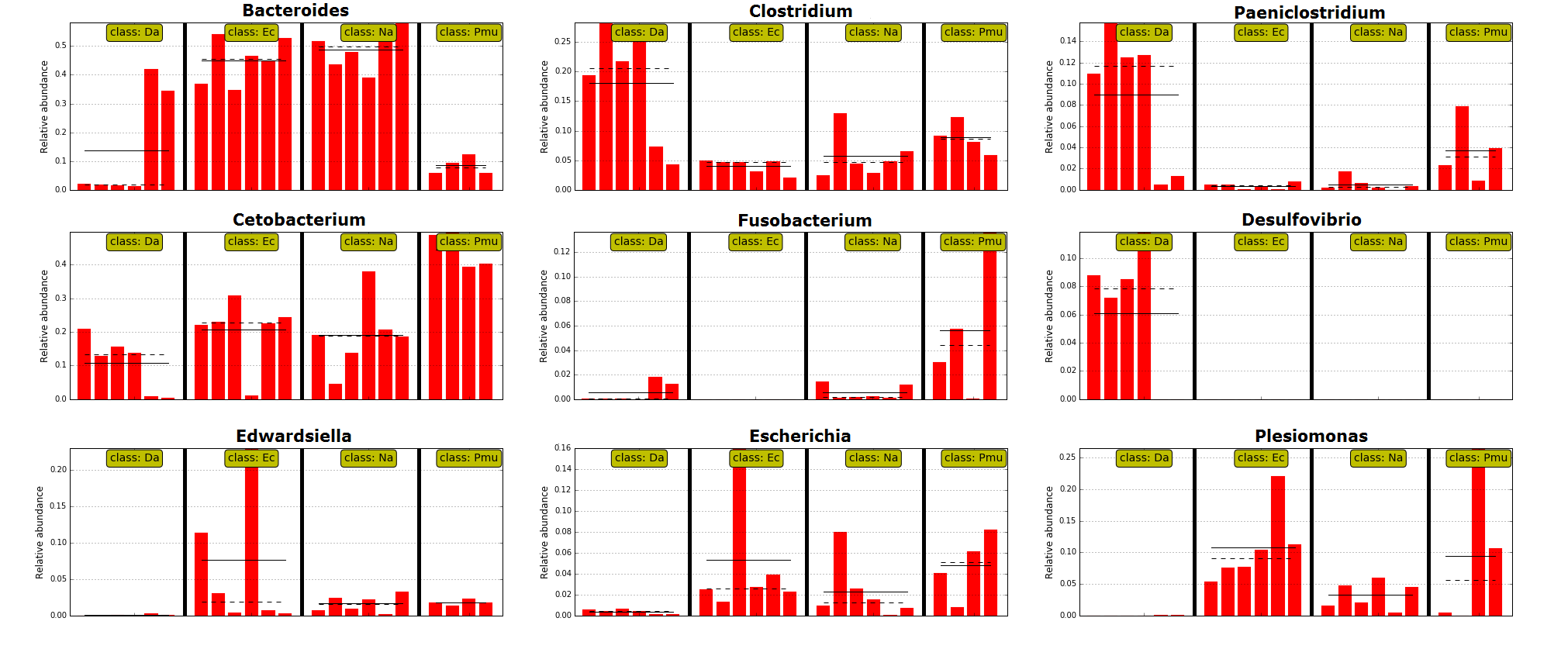

Supplement: Supplemental Information 4 — The taxa were selected by LEfSe analysis at the genus level. The straight line is the mean. The dot line is the median. Na, Naja atra group; Pmu, Ptyas mucosus group; Ec, Elaphe carinata group; Da, Deinagkistrodon acutus group. [file peerj-07-6658-s004.png]
