# Supplementary material for: Comparative analysis and characterization of the gut microbiota of four farmed snakes from southern China
Source: PeerJ. 2019 Mar 29;7:e6658. doi: 10.7717/peerj.6658 (PMC6442672; doi:10.7717/peerj.6658)
Supplement: Supplemental Information 5 — Da1-Da6 represent the samples collected from the Deinagkistrodon acutus, Ec1-Ec6 represent the samples collected from the Elaphe carinata, Na1-Na6 represent the samples collected from the Naja atra, Pmu1-Pmu6 represent the samples collected from the Ptyas mucosus. [file peerj-07-6658-s005.doc]

| Samples | Sequence number | OTUs number |
| --- | --- | --- |
| Da1 | 37005 | 294 |
| Da2 | 36035 | 305 |
| Da3 | 23898 | 264 |
| Da4 | 34607 | 306 |
| Da5 | 30010 | 270 |
| Da6 | 29635 | 293 |
| Ec1 | 33491 | 127 |
| Ec2 | 36166 | 101 |
| Ec3 | 34013 | 122 |
| Ec4 | 34104 | 97 |
| Ec5 | 36064 | 79 |
| Ec6 | 35432 | 122 |
| Na1 | 33165 | 160 |
| Na2 | 20934 | 104 |
| Na3 | 32888 | 184 |
| Na4 | 34074 | 193 |
| Na5 | 34124 | 123 |
| Na6 | 34184 | 161 |
| Pmu1 | 33358 | 167 |
| Pmu2 | 34705 | 163 |
| Pmu3 | 34681 | 130 |
| Pmu4 | 34737 | 113 |
